# An acute phase reaction from zoledronate mimicking symptoms seen in opioid withdrawal: a case report

**DOI:** 10.1186/s13722-024-00464-8

**Published:** 2024-05-01

**Authors:** Pankti P. Acharya, Crystal Joseph

**Affiliations:** 1 Department of Anesthesiology, Robert Wood Johnson University Hospital, New Brunswick, NJ USA; 2 Department of Anesthesiology, Beth Israel Deaconess Medical Center, Boston, MA USA

**Keywords:** Acute pain, Addiction medicine, Cognitive bias, Pain medicine, Zoledronic acid

## Abstract

**Background:**

Zoledronate, a bisphosphonate, is a potent first-line treatment for osteoporosis. It is also a preferred treatment for hypercalcemia especially when unresponsive to intravenous fluids. Bisphosphonates can cause acute phase reactions that mimic opioid withdrawal symptoms, which can confound provider decision-making. Our case highlights cognitive bias involving a patient with opioid use disorder who received zoledronate for hypercalcemia secondary to immobilization and significant bone infection.

**Case presentation:**

A 41-year-old male is admitted with a past medical history of active intravenous opioid use complicated by group A streptococcal bacteremia with L5-S1 discitis and osteomyelitis, L2-L3 osteomyelitis, and left ankle abscess/septic arthritis status post left ankle washout. His pain was well-controlled by acute pain service with ketamine infusion (discontinued earlier), opioids, acetaminophen, buprenorphine-naloxone, cyclobenzaprine, gabapentin, and naproxen. Intravenous opioids were discontinued, slightly decreasing the opioid regimen. A day later, the patient reported tachycardia, diaphoresis, myalgias, and chills, which the primary team reconsulted acute pain service for opioid withdrawal. However, the patient received a zoledronate infusion for hypercalcemia, on the same day intravenous opioids were discontinued. He had no other medications known to cause withdrawal-like symptoms per chart review. Therefore, it was suspected that an acute phase reaction occurred, commonly seen within a few days of bisphosphonate use.

**Conclusion:**

Zoledronate, well known for causing acute phase reactions, was likely the cause of withdrawal-like symptoms. Acute phase reactions with bisphosphonates mostly occur in the first infusion, and the incidence decreases with subsequent infusions. Symptoms typically occur 24–72 h post-infusion, and last at most for 72 h. Cognitive bias led the primary team to be concerned with opioid withdrawal rather than investigating other causes for the patient’s presentation. Therefore, providers should thoroughly investigate potential etiologies and rule them out accordingly to provide the best care. Health care providers should also be aware of the implicit biases that potentially impact the quality of care they provide to patients.

## Introduction

Zoledronate, a medication found in the bisphosphonate class, has been used to treat a variety of conditions, including osteoporosis, Paget disease and hypercalcemia. This medication works by strongly binding to hydroxyapatite in bone, leading to osteoclast destruction and decreases bone loss [1].

When given intravenously, bisphosphonates can lead to acute phase response-like symptoms, including fevers, myalgias, and fatigue. Typically, this reaction occurs in bisphosphonate naive patients following the first dose. It usually presents within 36 h of bisphosphonate administration and will diminish within a couple days [2]. Approximately 30–40% of individuals experience an acute phase response to an initial intravenous nitrogen-containing bisphosphonate dose [2, 3].

The Horizon-Pivotal Fracture study was a multicenter clinical trial studying intravenous zoledronic acid infusion in postmenopausal women with osteoporosis. Patients receiving the zoledronic acid infusion experienced significantly more fevers (17.2%; compared to placebo 1.8%), diffuse musculoskeletal pain (15.7%; compared to placebo 3.0%), and gastrointestinal symptoms (7.8%; compared to placebo 2.1%). Risk factors of acute phase responses include back pain, younger age, and recent NSAID use. Patients who were living with diabetes mellitus, smokers, or have previously used bisphosphonate or calcitonin were less likely to have an acute phase response [4].

Additionally, the type of bisphosphonate has been shown to have differences in the severity of acute phase reactions. For example, a recent study found a significant difference in the acute phase response following the first dose response of intravenous zoledronate (69.4%) compared to intravenous ibandronate (38.2%) in bisphosphonate naive individuals. Additionally, there was overall a greater incidence of an acute phase response following zoledronate use compared to ibandronate [5]. Prior to intravenous bisphosphonate use, additional testing should be completed, such as complete blood count, complete metabolic panel, and phosphate. After medication administration, clinically pertinent signs of hypocalcemia should be closely monitored [6].

## Case report

A 41-year-old male presented to our hospital with a past medical history of active intravenous opioid use. This was complicated by group A streptococcal bacteremia with L5-S1 discitis and osteomyelitis, L2-L3 osteomyelitis, and a left psoas abscess. Additional complications included a right-sided parapneumonic effusion with septic emboli and cavitary lesions, and left ankle abscess with septic arthritis status post a left ankle washout during this hospitalization. He was conservatively managed with intravenous antibiotics for his discitis and spine osteomyelitis as recommended by neurosurgery.

His opioid use history involves being treated with buprenorphine-naloxone, with several recovery periods throughout the year after starting the medication. Although he had previous episodes of recovery with the use of buprenorphine-naloxone, he had started using intravenous heroin for about 3 months before being admitted. He last used his buprenorphine-naloxone about 1 year prior. During his hospitalization, he was initiated on buprenorphine-naloxone 8 mg twice a day, which was modified to 4 mg sublingual twice a day and further tapered down to 2 mg twice a day after the patient was experiencing significant diaphoresis which he felt was from taking the buprenorphine-naloxone. The Acute Pain Service was consulted for optimization of his pain regimen due to his current infection, recent ankle washout, and prior opioid use history.

The patient describes his pain as deep, aching, and mainly located in the lower back and left ankle. His pain is aggravated by movement and improves with rest. His pain was well-controlled by the Acute Pain Service with a ketamine infusion that has since been discontinued earlier, opioid regimen as needed, acetaminophen, buprenorphine-naloxone, cyclobenzaprine, gabapentin, and naproxen (Table 1). He states that his pain has overall been more manageable aside from exacerbations with movement. On further conversation regarding his pain regimen, the patient agreed on the goal of weaning and discontinuing intravenous opioids. Intravenous opioids were discontinued, slightly decreasing the opioid regimen the patient was on.

**Table 1:** Inpatient multimodal analgesic regimen in a 41-year-old male with an opioid withdrawal-like acute phase reaction from intravenous zoledronate

Day 1 (prior to symptom onset) *Zoledronate Infusion Given	Day 2 (symptom onset)	Day 3 (symptom resolution)
⋅ Discontinued intravenous hydromorphone 0.5 mg every 6 h as needed for breakthrough pain ⋅ Acetaminophen 975 mg every 8 h scheduled ⋅ Buprenorphine-Naloxone 2 mg twice daily ⋅ Oral hydromorphone 4 and 6 mg every 3 h as needed for moderate to severe pain ⋅ Cyclobenzaprine 5 mg 3 times daily ⋅ Gabapentin 600 mg 3 times daily ⋅ Naproxen 500 mg twice daily	⋅ Acetaminophen 975 mg every 8 h scheduled ⋅ Buprenorphine-Naloxone 2 mg twice daily with plan to up titrate to minimum dose of 8 mg total daily dose prior to discharge ⋅ Oral hydromorphone 4 and 6 mg every 3 h as needed for moderate to severe pain ⋅ Cyclobenzaprine 5 mg 3 times daily ⋅ Gabapentin 600 mg 3 times daily ⋅ Naproxen 500 mg twice daily	⋅ Same as Day 2

One day later, the patient was noted to be experiencing tachycardia, diaphoresis, myalgias, hypertension, and chills, which the primary team reconsulted the Acute Pain Service to re-evaluate for opioid withdrawal. The patient felt his pain did not escalate or worsen in the twenty-four hours since the Acute Pain Service team evaluated him. He still described his pain as deep, aching in the lower back and left ankle, but was still manageable and slowly improving. He remained afebrile with a max temperature of 99.7 degrees Fahrenheit. Laboratory studies showed no leukocytosis. We did not make any changes to his opioid regimen for the day.

Upon evaluation of the medication regimen changes, the total daily opioid dosing was decreased slightly, and it was very unlikely that there was any level of opioid withdrawal with the minimal changes to the patient’s medication regimen (Table 2). Worsening infection was another differential to consider given the patient’s presentation of discitis, osteomyelitis, abscess, and septic arthritis. However, the patient was afebrile and his max temperature was 99.7 degrees Fahrenheit. He did not have an elevated white blood cell count, so this was an unlikely reason for the patient’s symptoms. Rebound pain was considered however, the patient felt his pain was manageable and did not worsen even with the de-escalation of his pain regimen. However, the patient received zoledronate infusion therapy for hypercalcemia on the same day intravenous opioids were discontinued. He initially received an intravenous infusion of 0.9% sodium chloride for hypercalcemia of 12.3 mg/dl (Institution Reference Range 8.4–10.2 mg/dl), which was suspected to be from prolonged immobilization in the setting of severe bone infection. The patient’s serum creatinine was 0.9 (Institution Reference Range 0.6–1.3 mg/dl) before intravenous zolendronate use. However, his serum calcium level did not improve, so the patient was given a one-time dose of 4 mg of intravenous zoledronate. He had no other medications known to cause withdrawal-like symptoms per chart review. Therefore, it was suspected that an acute phase reaction occurred, which is commonly seen within a few days of bisphosphonate use, especially with an intravenous infusion.

**Table 2:** 24-h opioid requirements by day in a 41-year-old male with a history of opioid use disorder complicated by significant infection hospitalized for treatment and surgical intervention

Days	24 Hour Opioid Requirement	Morphine Milligram Equivalents (MME)
Day 1 (prior to symptom onset) *Zoledronate Infusion Given*	1. 36 mg oral hydromorphone 2. 0.5 mg intravenous hydromorphone 3. 4 mg sublingual buprenorphine-naloxone	146 MME
Day 2 (symptom onset)	1. 30 mg oral hydromorphone 2. 4 mg sublingual buprenorphine-naloxone	120 MME
Day 3 (symptom resolution)	1. 30 mg oral hydromorphone 2. 4 mg sublingual buprenorphine-naloxone	120 MME

## Discussion

Bisphosphonates are well-known for causing acute phase reactions, especially in intravenous formulations [7]. Acute phase reactions can cause transient symptoms similar to opioid withdrawal, such as headache, malaise, myalgias, arthralgias [4]. Acute phase reactions with bisphosphonates mostly occur with or after first infusion, and its incidence decreases with subsequent infusions [7]. Symptoms typically occur 24–72 h post-infusion, and last at most for 72 h. Zoledronate is the most potent and long-acting bisphosphonate in the class of medications [8]. Its most common side effect is the acute phase reaction, seen in about 30 to 40% of patients after the first dose. Case reports have demonstrated the acute phase reaction that is experienced after receiving intravenous zoledronate [9–11].

We believe that our patient experienced an acute phase reaction after receiving intravenous zoledronate for treatment of his hypercalcemia, and the primary team incorrectly thought that the patient was experiencing an opioid withdrawal episode despite minimal change to his pain regimen. Our case highlights the importance of considering all possible differentials and deciding which is most likely causing the patient’s presentation. Failure to do so can cause an incorrect diagnosis to be made and inappropriate treatment for the patient, leading to either erroneous treatment, medical error, patient dissatisfaction, or an adverse event. With certain populations, especially those with a substance use disorder, the social stigmas and assumptions can influence providers to be more likely to lean on cognitive biases to determine patient diagnoses and treatments. However, this is a disservice to providing optimal patient care, and it is vital that as providers, we acknowledge our biases and aim to objectively care for our patients as much as possible. Providers should thoroughly investigate potential etiologies and rule out accordingly to provide the best individualized care.

Through a retrospective analysis of a large hospital, Keister et al. found that pain was being undertreated due to multiple underlying factors, such as stereotypes, subjective measures of pain assessment, and institutional policies. Due to their own implicit biases, certain physicians assume that certain races or genders have a higher pain tolerance or might be falsifying symptoms to seek pain medication [12]. This study highlights the presence of various cognitive biases that can impact patient care.

There are several assessments to evaluate for bias among healthcare workers. For example, the Implicit Association Test (IAT) analyzes attitudes or stereotypes through various questions [13, 14]. Similarly, Kennedy-Hendricks et al. created a web based survey that was distributed among the public to assess stigma towards patients with opioid use disorder and understand how to structure policy initiatives [15].

Stigma towards patients who have substance use disorder can be extremely isolating, shameful, and even affect the physican-patient relationship. Several studies have been conducted to identify interventions to improve patient care and decrease stigma towards people with substance use disorder. Chapman et al. suggests several strategies to combat implicit bias, such as individuating and perspective taking. Individuating focuses on that specific person’s symptoms and history, rather than grouping them based on their medical history, race, or gender [13]. Additionally, perspective taking allows for the provider to put themselves in the patient’s situation. Similarly, Drwecki et al. found by utilizing an empathy based treatment approach, there was a 55% increase in the treatment of pain among nurses compared to a control group [16].

Bielenberg et al. conducted a systematic review of interventions to combat stigma towards patients with substance use disorder. There are various online and in-person educational training sessions offered. For example, an in-person educational workshop educated healthcare providers on harm reduction, training for crisis, and providing them with tools to speak with patients who have substance use disorders. Following these workshops, there was a significant improvement in knowledge and improvement in their role [17, 18]. Many of these interventions included stories of patients who are experiencing substance use disorder and described their struggles. After understanding this, there was an improvement of attitudes among providers. For example, a study utilized a short online module to educate residents physicians. Following this module, there was a significant improvement among residents about patients with substance use disorder even several months after the intervention [17, 19].

In conclusion, stigma towards patients with substance use disorder can affect clinical judgement. It is important to identify implicit biases and address them on an individual and systemic level.

## Data Availability

Not applicable.
